# Supplementary material for: Enrofloxacin Alters Fecal Microbiota and Resistome Irrespective of Its Dose in Calves
Source: Microorganisms. 2021 Oct 17;9(10):2162. doi: 10.3390/microorganisms9102162 (PMC8537546; doi:10.3390/microorganisms9102162)
Supplement: Supplementary file 1 [file microorganisms-09-02162-s001.zip › microorganisms-1409520-supplementary.pdf]

microorganisms

MDPI

Supplementary Materials

Figure S1: Ten most abundant phyla by groups (top) and sampling days (bottom, enrofloxacin was administered to the calves on day 21 right after sample collection)

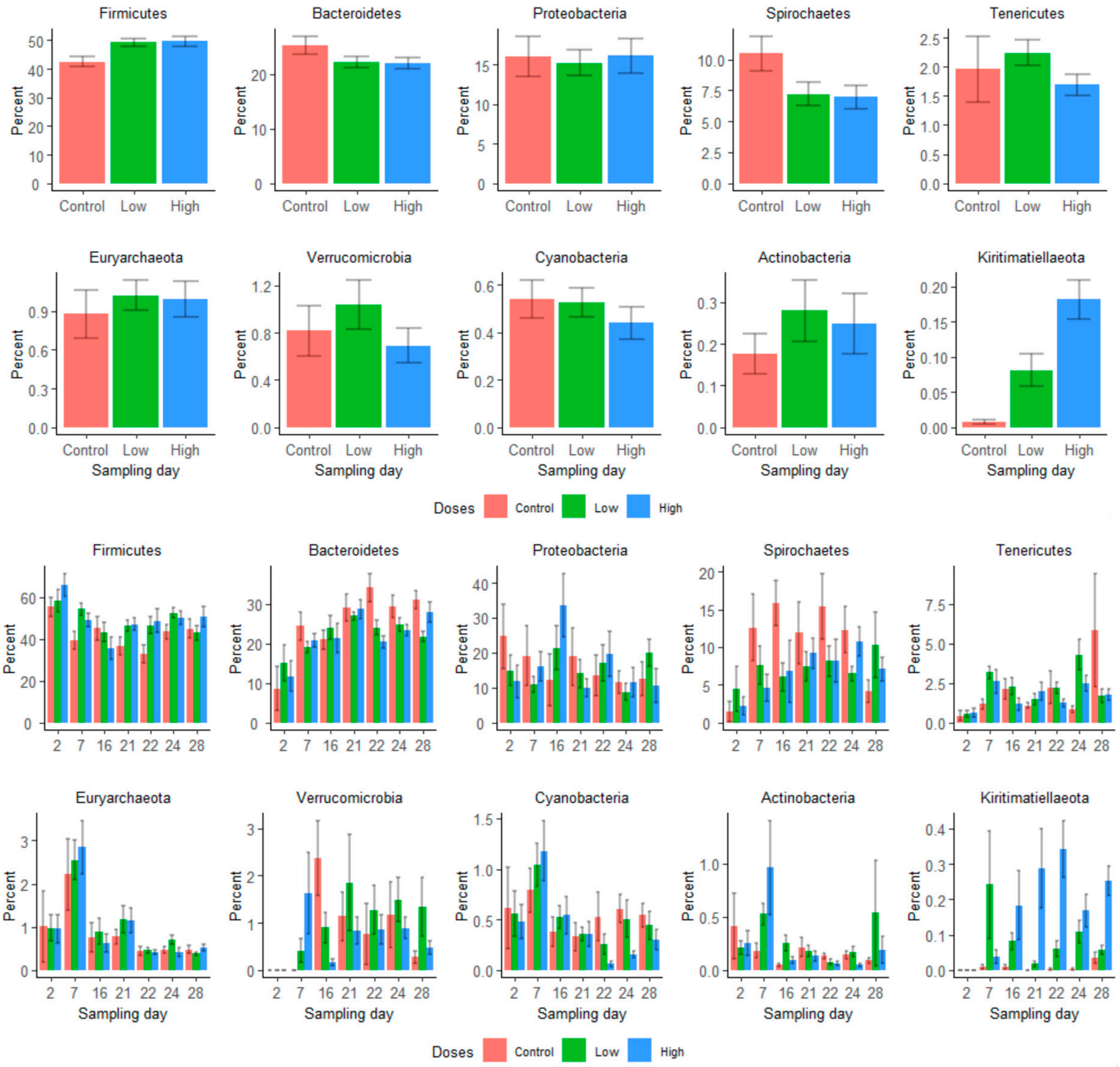

Table S1. Core members of fecal microbiota in beef calves (≥90% samples, total = 245)

Phylum	Class	Order	Family	genus	Number of samples
Euryarchaeota	Methanobacteria	Methanobacteriales	Methanobacteriaceae	Methanobrevibacter	222
Bacteroidetes	Bacteroidia	Bacteroidales	Bacteroidaceae	Bacteroides	227
Bacteroidetes	Bacteroidia	Bacteroidales	Muribaculaceae	uncultured	220
Bacteroidetes	Bacteroidia	Bacteroidales	Prevotellaceae	Alloprevotella	222
Bacteroidetes	Bacteroidia	Bacteroidales	Prevotellaceae	Prevotellaceae_UCG-003	223
Bacteroidetes	Bacteroidia	Bacteroidales	Rikenellaceae	Alistipes	222
Bacteroidetes	Bacteroidia	Bacteroidales	Rikenellaceae	Rikenellaceae_RC9_gut_group	230
Firmicutes	Clostridia	Clostridiales	Clostridiaceae_1	Clostridium_sensu_stricto_1	225
Firmicutes	Clostridia	Clostridiales	Ruminococcaceae	uncultured	220
Firmicutes	Clostridia	Clostridiales	Clostridiales_vadinBB60_group	uncultured	225
Firmicutes	Clostridia	Clostridiales	Lachnospiraceae	Unassigned	243
Firmicutes	Clostridia	Clostridiales	Ruminococcaceae	Ruminococcaceae_UCG-005	235
Firmicutes	Clostridia	Clostridiales	Ruminococcaceae	Ruminococcaceae_UCG-010	225
Firmicutes	Clostridia	Clostridiales	Ruminococcaceae	Ruminococcaceae_UCG-014	223
Firmicutes	Clostridia	Clostridiales	Ruminococcaceae	[Eubacterium]_coprostanoligenes_group	235
Firmicutes	Clostridia	Clostridiales	Ruminococcaceae	Unassigned	237
Proteobacteria	Gammaproteobacteria	Betaproteobacteriales	Burkholderiaceae	Sutterella	221
Spirochaetes	Spirochaetia	Spirochaetales	Spirochaetaceae	Treponema_2	230

Table S2: Analysis of composition of microbiomes (ANCOM) comparing pre-and post-treatment samples at the genus level.

	Genus	Pre-treatment					Post-treatment				
		0	25	50	75	100	0	25	50	75	100
1	Actinobacteria;Actinobacteria;Bifidobacteriales;Bifidobacteriaceae;Bifidobacterium	1	1	9*	47	1057	1	1	1	1	703
2	Bacteroidetes;Bacteroidia;Bacteroidales;Bacteroidaceae;Bacteroides	1	188	711	1270	3832	65	731	1070	1479	3162
3	Bacteroidetes;Bacteroidia;Bacteroidales;Bacteroidales_RF16_group;__	1	1	1	1	2036	1	1	15	219	1939
4	Bacteroidetes;Bacteroidia;Bacteroidales;Bacteroidales_RF16_group;uncultured_bacterium	1	9	92	337	2602	7	64	247	523	2623
5	Bacteroidetes;Bacteroidia;Bacteroidales;Bacteroidales_UCG-001;uncultured_rumen_bacterium	1	1	1	1	38	1	1	1	5	560
6	Bacteroidetes;Bacteroidia;Bacteroidales;p-251-o5;uncultured_Bacteroidales_bacterium	1	1	1	21	756	1	13	42	111	1629
7	Bacteroidetes;Bacteroidia;Bacteroidales;Prevotellaceae;Prevotellaceae_UCG-001	1	1	22	83	682	1	36	61	115	417
8	Bacteroidetes;Bacteroidia;Bacteroidales;Prevotellaceae;Prevotellaceae_UCG-003	1	28	151	391	1517	73	303	484	758	1586
9	Bacteroidetes;Bacteroidia;Bacteroidales;uncultured;uncultured_bacterium	1	1	1	79	747	1	1	147	410	843
10	Bacteroidetes;Bacteroidia;Bacteroidales;uncultured;uncultured_Bacteroidales_bacterium	1	1	1	1	1615	1	1	1	322	1161
11	Epsilonbacteraeota;Campylobacteria;Campylobacterales;Campylobacteraceae;Campylobacter	1	1	1	7	104513	1	1	1	1	42
12	Firmicutes;Clostridia;Clostridiales;Clostridiales_vadinBB60_group;Ambiguous_taxa	1	1	1	7	1405	1	1	1	1	6
13	Firmicutes;Clostridia;Clostridiales;Lachnospiraceae;[Eubacterium]_hallii_group	1	1	1	8	118	1	1	23	35	85
14	Firmicutes;Clostridia;Clostridiales;Lachnospiraceae;Acetitomaculum	1	1	15	41	255	1	21	49	118	719
15	Firmicutes;Clostridia;Clostridiales;Lachnospiraceae;Lachnospiraceae_FCS020_group	1	1	13	27	94	1	20	40	67	195
16	Firmicutes;Clostridia;Clostridiales;Ruminococcaceae;Ruminiclostridium_1	1	1	1	6	87	1	1	11	22	246
17	Firmicutes;Clostridia;Clostridiales;Ruminococcaceae;Ruminococcaceae_UCG-002	1	14	111	214	1478	1	146	301	524	1122
18	Firmicutes;Clostridia;Clostridiales;Ruminococcaceae;Ruminococcaceae_UCG-005	1	1010	2502	4393	13036	433	2666	4145	5139	11420
19	Firmicutes;Clostridia;Clostridiales;Ruminococcaceae;Ruminococcaceae_UCG-010	1	147	768	1905	4442	47	905	1991	2883	5019
20	Kiritimatiellaeota;Kiritimatiellae;WCHB1-41;uncultured_rumen_bacterium;uncultured_rumen_bacterium	1	1	1	18	764	1	9	24	64	297
21	Proteobacteria;Alphaproteobacteria;Rhodospirillales;uncultured;__	1	1	10	40	503	1	1	1	9	107
22	Proteobacteria;Alphaproteobacteria;Rhodospirillales;uncultured;uncultured_bacterium	1	1	12	50	1060	1	1	1	1	242
23	Proteobacteria;Gammaproteobacteria;Enterobacteriales;Enterobacteriaceae;Escherichia-Shigella	1	1	10	32	64112	1	1	1	10	286
24	Spirochaetes;Spirochaetia;Spirochaetales;Spirochaetaceae;Treponema_2	1	74	444	2125	19561	1	646	1543	3347	15725
25	Tenericutes;Mollicutes;Anaeroplasmatales;Anaeroplasmataceae;Anaeroplasma	1	1	4	16	2974	1	7	20	64	3220
26	Tenericutes;Mollicutes;EMP-G18;uncultured_bacterium; uncultured_bacterium	1	1	4	20	274	1	10	27	49	317
27	Tenericutes;Mollicutes;Izimaplasmatales;gut_metagenome;gut_metagenome	1	1	1	1	152	1	1	1	60	666

28	Verrucomicrobia;Verrucomicrobiae;Verrucomicrobiales;Akkermansiaceae;Akkermansia	1	1	1	69	3380	1	1	90	436	2450

* 50th percentile (median) - in half of the pre-treatment samples, nine or fewer sequences were observed that were ultimately assigned *Bifidobacterium* compared to only one read assigned *Bifidobacterium* in the post-treatment samples.

Table S3. Reservoirs of antimicrobial resistance genes detected in pooled samples – control group

**Bacterial taxa**		**Pre-treatment**	**Post-treatment**
**Phylum**	**Low level**		
Actinobacteria	*Streptomyces_mobaraensis*_NBRC_13819_DSM_40847	NA*	tetW(1**)
Bacteroidetes	*Bacteroides_salanitronis*_DSM_18170	tet40(1), tetQ(1)	NA
	*Prevotella_stercorea*_DSM_18206	aph2(2), aph3(4), ant6(15), ant9(2), rob(2), ermB(1), ermF(1), ermG(2), ermQ(1), mefE(442), cfr(12), tet40(10), tetBP(1), tetO(4), tetQ(440), tetW(10)	aph2(1), aph3(1), ant6(10), ant9(2), ermF(11), mefE(106), cfr(10), tetW(1)
Firmicutes	*Eubacterium_siraeum*_CAG_80	tet32(1), tet40(29), tetW(12)	NA
	*Oscillibacter*_sp.	aph2(2), aph3(2), mefE(1), tetQ(4), tetW(1)	NA
	Streptococcus_suis_SS12	tet40(1), tetQ(3)	NA
Fusobacteria	*Fusobacterium_nucleatum*_subsp._vincentii_3_1_36A2	NA	tetA(1), tetW(1)
Proteobacteria	*Rhodopseudomonas_palustris*_DX_1	ermG(1), ermQ(1), mefE(1), tet40(1), tetQ(4)	ant6(1)
Spirochaetes	*Treponema*_sp.	cfr(1), tet40(8), tetW(2)	NA
	*Treponema_succinifaciens*_DSM_2489	NA	aph2(1), aph3(1), ant9(1), aci(2), cfr(22), tet40(46), tetW(13)

*NA - not available, this bacterial taxon was not detected, **number of hits.

Table S4. Reservoirs of antimicrobial resistance genes detected in pooled samples – low dose healthy group

Bacterial taxa		Pre-treatment	Post-treatment
**Phylum**	**Low-level**		
Actinobacteria	*Actinomyces_timonensis*_DSM_23838	NA*	tetW(3**)
	*Bifidobacterium_pseudolongum*_AGR2 145	aph2(2), aph3(2), ant6(20), ant9(1), ermF(765), mefE(3), sat(2), tet40(4), tetQ(1364), tetW(9)	NA
	Streptomyces_resistomycificus	NA	tet40(1)
Bacteroidetes	*Bacteroides*_sp._CAG_927	NA	tetQ(1)
	*Prevotella*_sp._CAG	aph2(2), aph3(3), ant6(13), ant9(2), rob(1), ermF(2), ermG(2), mefE(428), cfr(17), sat(2), tet40(4), tetM(1), tetO(1), tetQ(335), tetW(6)	No ARG
	*Prevotella_stercorea*_DSM_18206	NA	aph2(1), aph3(24), ant6(2), rob(24), ermG(1), mefE(83), tetQ(61), tetW(2), tetX(2)
Firmicutes	*Firmicutes*_bacterium_CAG_176	NA	tetW(99)
	*Blautia_producta*_ATCC_27340_DSM _2950	NA	tetO(1)
	*Clostridium*_sp._CAG_710	NA	ermQ(1), tet44(1), tetO(1) tetW(1)
	*Eubacterium*_sp._CAG_202	NA	tetO(5)
	*Paenibacillus_polymyxa*_M1	NA	tetW(1)
	*Roseburia*_sp._CAG_303	NA	tetW(1)
	*Ruminococcus*_sp._A254.MGS_108	NA	tetW(7)
Proteobacteria	*Enterobacter*_sp._R4_368	tetQ(3)	NA
	*Erythrobacter_litoralis*_HTCC2594	NA	tet32(2), tet40(2)
	*Halomonas*_sp._PBN3	NA	tetW(2)
	*Nitrosococcus_halophilus*_Nc_4	NA	tet(15), tetW(1)
	*Ralstonia_solanacearum*_Po82	tet40(2), tetQ(3), tetW91)	NA
	*Rhodopseudomonas_palustris*_DX_1	NA	aph3(1), rob(1), tetQ(4)
Spirochaetes	*Treponema*_sp._JC4	aph2(6), aph3(6), ant6(6), aci(15), ermF(1), cfr(10), sat(6), tet40(57), tetM(1), tetO(28), tetW(122)	tet40(6), tetW(3)
Tenericutes	*Mycoplasma* sp.	NA	cfr(1), tet40(7), tetW(2)

*NA – not available, this bacterial taxon was not detected, **number of hits.

Table S5. Reservoirs of antimicrobial resistance genes detected in pooled samples – high dose healthy group

**Bacterial taxa**		**Pre-treatment**	**Post-treatment**
**Phylum**	**Low level**		
Bacteroidetes	*Bacteroides*_sp._CAG_770	ant6(1), mefE(3), tet40(1), tetW(2)	NA
	*Bacteroides_coprophilus*_DSM_18228_JCM_13818	aph2(12), aph3(12), ant6(27), ant9(4), ermG(2), ermQ(3), mefE(10771), cfr(25), sat(3), sulII(1), tet32(1), tet40(35), tet44(5), tetA(5), tetB(12), tetM(1), tetO(7), tetQ(577), tetW(41), tetX(1)	NA
	*Bacteroides_fragilis*_str._3725_D9_v_.	ant6(1), tetW(1)	NA
	*Phocaeicola_abscessus*_CCUG_55929	mefE(6), tet40(1), tetA(1), tetB(1), tetO(2), tetQ(2), tetW(1)	NA
	*Prevotella*_sp._P5_119	NA	aph2(15), aph3(15), ant9(128), ermG(62), cfr(1), tet40(1), tetO(1), tetW(9)
	*Prevotella*_sp._oral_taxon_299_str._F0039	NA	mefE(1), tet40(1), tetW(2)
	*Prevotella_intermedia*_17	tetA(1), tetW(1)	NA
	*Prevotella_ruminicola*_23	NA	ant6(2), ant9(1), oxa(2), cfr(1), tet40(1), tetW(10)
	*Prevotella_stercorea*_DSM_18206	NA	aph2(3), aph3(4), ant6(10), ant9(7), rob(1), ermF(1), ermG(1), ermQ(1), mefE(506), cfr(9), sat(3), tet32(1), tet40(10), tetA(1), tetO(1), tetW(78), tetX(1)
Chlamydia	*Chlamydia_pecorum*_PV3056_3	mefE(2),	NA
Cyanobacteria	*Cyanobacterium_aponinum*_PCC_10605	NA	aph2(1), aph3(1), ant6(1), tet40(10), tetW(10)
Firmicutes	*Carboxydothermus_hydrogenoformans*_Z_2901	aph2(4), aph3(4), ant9(2), mefE(5), tet40(3), tetB(2), tetQ(1), tetW(2)	NA
	*Clostridium*_sp._CAG_914	ant6(1), ermQ(1), mefE(9), sat(2), tet40(2), tet44(9), tetA(2), tetB(3), tetM(1), tetO(1), tetW(2)	NA
	*Eubacterium*_sp._CAG_161	NA	sat(1), tet40(2), tetO(2), tetW(81)
	*Eubacterium_ventriosum*_ATCC_27560	aph2(4), aph3(4), ant6(2), ermQ(1), mefE(22), tet40(5), tet44(1), tetB(1), tetO(27), tetQ(4), tetW(5)	NA
	Clostridiales	aph2(441), aph3(441), ant6(9), ant9(18), ermB(1), ermG(2), mefE(55), sat(82), tet40(306), tetA(6), tetB(11), tetM(2), tetO(6), tetQ(7), tetW(366)	aph2(1), aph3(1), oxa(160), sat(1), tet32(2), tet40(3), tetO(5), tetW(318)
	*Ruminococcus*_sp._CAG_488	aph2(2), aph3(2), ant6(1), mefE(6), cfr(1), sat(1), floR(1), tet40(11), tetA(1), tetO(7), tetQ(1), tetW(4)	aph2(3), aph3(3), ant6(1), mefE(1), tet40(18), (tetW(23)
	*Subdoligranulum*_sp._4_3_54A2FAA	aph2(16), aph3(16), ant6(2), mefE(13), sat(11), tet40(3), tet44(1), tetB(4), tetO(2), tetQ(2), tetW(25)	NA
Euryarchaeota	*Methanobrevibacter_oralis*_JMR01	ant6(1), ant9(1), mefE(6), tet40(1), tet44(1), tetW(1)	aph2(3), aph3(4), ant6(1), rob(1), tet40(6), tetO(1), tetW(26)

	*Methanobrevibacter_ruminantium*_M1	NA	sat(1), tet40(1), tetW(13)
	*Methanobrevibacter_wolinii*_SH	mefE(10), tet40(1), tetB(2), tetM(1), tetO(1), tetW(2)	ant6(1), tetW(1)
Proteobacteria	*Rhodopseudomonas_palustris*_DX_1	aph2(2), aph3(2), mefE(40), tet40(2), tetB(3), tetQ(13), tetW(5)	aph291), aph3(1), tetW(3)
	*Rickettsia_heilongjiangensis*_054	NA	tetW(6)
Spirochaetes	*Treponema*_sp._JC4	NA	cfr(2), tet32(1), tet40(7), tetO(1), tetW(7)
	*Treponema_succinifaciens*_DSM_2489	NA	tet32(8), tet40(14), tetO(3), tetW(18)
Tenericutes	*Mycoplasma* sp.	aph2(1), aph3(1), mefE(12), cfr(19), tet40(21), tetA(1), tetB(1), tetO(2), tetW(13)	NA
Verrucomicrobia	*Akkermansia_muciniphila*_ATCC_BAA_835	aph2(7), aph3(8), ant9(5), rob(1), ermB(1), ermG(1), ermQ(1), ermX(1), mefE(275), sat(7), tet32(1), tet40(39), tet44(2), tetA(12), tetB(26), tetM(2), tetO(10), tetQ(11), tetW(60)	aph2(1), aph3(1), ant6(1), ermF(1), sat(1), tet32(1), tet40(10), tetA(1), tetO(2), tetW(30), tetX(1)
Other	*Candidatus_Liberibacter_americanus*_str._Sao_Paulo	mefE(5), tet40(2), tetO(1)	NA

*number of hits

Table S6. Reservoirs of antimicrobial resistance genes detected in pooled samples – high dose sick group

Bacterial taxa		Pre-treatment	Post-treatment
**Phylum**	**Low level**		
Actinobacteria	*Corynebacterium_nuruki*_S6_4	cfr(2), tetO(2), tetW(7)	ermQ(1), mefE(6), tetW(2)
Bifidobacteria	*Bifidobacterium_pseudolongum*_subsp._globosum_DSM_20092	NA	ant6(1), cmX(4), ermQ(1), ermX(2), mefE(4), sat(1), tet40(1), tetA(1), tetQ(1), tetW(1)
Bacteroidetes	*Bacteroides*_sp.	aph2(1), aph3(1), ant6(5), cfX(2), mefE(1748), cfr(3), sat(1), tet40(1), tetQ(13), tetW(1)	aph2(1), aph3(1), tet40(1), tetM(2), tetQ(1)
	*Prevotella*_sp._CAG_5226.fna	NA	mefE(1), tetO(1)
	*Prevotella_scopos*_JCM_17725.fna	NA	aph2(1), aph3(1), ant6(1), ant9(1), mefE(6), tet40(3), tetA(1), tetQ(6), tetW(2)
	*Prevotella_stercorea*_DSM_18206	NA	aph2(5), aph3(5), ant6(18), ant9(16), cmX(3), ermG(1), ermQ(6), mefE(4606), cfr(12), sat(1), tet40(20), tetA(7), tetB(1), tetM(1), tetO(3), tetQ(391), tetW(49)
Firmicutes	*Bacillus_megaterium*_QM_B1551	NA	mefE(4), tet40(2), tetO(7), tetQ(1), tetW(5)
	*Butyrivibrio_proteoclasticus*_B316	NA	aph2(1), mefE(1)
	*Clostridium*_sp._CAG_710.fna	tetO(21)	ant6(3), mefE(1), tetW(3)
	*Clostridium_aminophilum*_DSM_10710	NA	mefE(2), tet40(12), tetQ(1), tetW(3)
	*Eubacterium_rectale*_ATCC_33656	NA	mefE(3), tet40(3), tetA(1)
	*Geomicrobium*_sp._JCM_19055	NA	tetW(5)
	*Streptococcus_pneumoniae*_ST556	NA	mefE(2), tetO(3), tetW(14)
	*Streptococcus_pyogenes*_MGAS1882	NA	aph2(5), aph3(3), mefE(1), tet40(16)
	*Streptococcus*_suis_D9	NA	ermQ(1), mefE(1), tetW(3)
	*Subdoligranulum*_sp._4_3_54A2FAA	aph2(5), aph3(5), ant6(2), sat(5), tet40(1), tetW(4)	NA
Nitrospirae	*Thermodesulfovibrio_yellowstonii*_DSM_11347	NA	mefE(1), tet40(2), tetW(1)
Proteobacteria	*Arcobacter_butzleri*_7h1h	NA	ermF(1), mefE(1), tet40(4), tetX(1)
	*Erythrobacter_litoralis*_HTCC2594	NA	ant6(2), ant9(2), mefE(1), tet40(1), tetW(1)
Spirochaetes	*Treponema_succinifaciens*_DSM_2489	NA	aci(1), ermQ(1), mefE(4), cfr(16), tet40(27), tet44(1), tetQ(1), tetW(16)

*number of hits
